# A pilot study of research methods for determining the impact of pictorial cigarette warning labels among smokers

**DOI:** 10.1186/1617-9625-12-16

**Published:** 2014-09-24

**Authors:** Darren Mays, Sarah E Murphy, Andrea C Johnson, John D Kraemer, Kenneth P Tercyak

**Affiliations:** 1 Department of Oncology, Georgetown University Medical Center, Lombardi Comprehensive Cancer Center, 3300 Whitehaven St NW, Suite 4100, Washington DC 20007, USA

**Keywords:** Pictorial cigarette warning labels, Cigarette smoking, Young adults

## Abstract

**Background:**

Science to determine the impact of pictorial cigarette warning labels can inform decisions about warning label implementation and adjustments to their contents to maximize impact. This pilot study builds from earlier research on plain cigarette packaging to examine the feasibility of a method for determining the impact of pictorial warnings among smokers.

**Findings:**

The study was a prospective, within-subjects pilot trial where smokers ages 18–30 (*n* = 10) were exposed to pictorial warnings on their cigarette packs. On day one, participants completed a baseline interview with an expired carbon monoxide reading and affixed pictorial warning labels to their cigarette pack(s) they would use the next day. On day two, participants completed mobile phone text message assessments of smoking behaviors and protocol adherence. On day three, participants completed a follow-up interview similar to baseline. We achieved 100% sample retention and adherence with procedures. Compared with baseline assessments of perceptions and behaviors related to existing text-only warnings, at follow-up participants were more likely to report that pictorial warnings used during the study were noticeable (*M* 4.1, *SD* 1.3 vs. *M* 2.7, *SD* 1.2, *p* = .013), stopped them from smoking (*M* 1.6, SD 0.8 vs. *M* 1.1, *SD* 0.3, *p* = .052), and conveyed health risks of smoking (*M* 3.5 *SD* 1.3 vs. *M* 2.2, SD 1.1, *p* = .006). At follow-up, participants also reported the protocol was acceptable.

**Conclusions:**

These findings suggest this is a feasible method that with further validation could provide evidence that can inform decisions regarding implementation of pictorial cigarette warnings.

## Findings

### Background

Article 11 of the World Health Organization’s Framework Convention on Tobacco Control (FCTC) recommends strong pictorial warnings for cigarette packages as part of a comprehensive policy approach for tobacco control [1]. Globally, more than 60 countries have adopted pictorial warning labels as a tobacco control policy [2,3]. Research investigating the impact of pictorial warnings consists primarily of population-based surveys examining self-reported outcomes surrounding warning labels, or studies using methods such as online experiments to examine the impact of warnings following a brief exposure [1,3,4]. Although this research supports the use of pictorial warnings as a tobacco control measure, additional research can inform decision-making about cigarette warnings in a number of settings that are at various stages in the process of their implementation.

For example, in the U.S. the 2009 Family Smoking Prevention and Tobacco Control Act required that new pictorial warning labels replace existing text only warnings on cigarette packs. However, tobacco industry lawsuits have delayed their implementation, with recent court rulings citing in part insufficient evidence of their impact [5,6]. This signifies a need for research that can better address the judicial concerns surrounding implementing the pictorial warnings in this context. In addition, in many countries where pictorial warnings have already been adopted, a challenge public health officials face is how to adapt the contents of warnings over time for sustained effectiveness [7]. Research examining the optimal contents of warning label messages can inform such decisions in these settings as well.

A behavioral epidemiology framework characterizing the phases of prevention science can be adapted and applied to advance research on pictorial warnings [8,9]. “Phase 1” studies identify associations between an exposure (e.g., pictorial warnings) and relevant disease prevention outcomes (e.g., intention or attempts to quit smoking). Many studies of pictorial warnings conducted to date fall within this phase. “Phase 2” studies seek to pilot test and validate new methods for determining the impact of an exposure on behavioral outcomes [8]. “Phase 2” studies provide a methodological foundation for experimental and population-based research to determine an intervention’s impact and to translate findings into policy [8,9], two fundamental goals of tobacco regulatory science [10].

Guided by this framework, we conducted a “Phase 2” pilot study to examine the feasibility of a research protocol using multiple behavioral assessment modalities for determining the impact of pictorial cigarette warning labels among young smokers. This pilot study builds from prior research examining plain cigarette packaging that provides a model for rigorous, trial-like methods for understanding the impact of cigarette packaging that can be extended to study pictorial cigarette warning labels [11-13]. The results of this pilot study can lay the groundwork for future research to determine the impact of pictorial warning labels and other cigarette packaging regulations on cigarette smoking behaviors, such as cigarettes smoked, quit attempts, and cessation.

## Methods

Ten young adults ages 18 to 30 who smoked ≥ 100 lifetime cigarettes and currently smoked on all or some days were recruited to participate in a within-subjects prospective pilot trial. On day one, participants completed a baseline interview measuring demographics, cigarette smoking behaviors and motivation to quit smoking [14,15]. Measures captured participants’ perceptions of warnings (warnings are noticeable, serious, believable, convey risks) using four items with a five point Likert-type response and behaviors in response to warnings (noticed warnings, read warnings, warnings stopped them from smoking) using 3 items with a similar response scale. These items were derived from prior studies [16,17]. At baseline the items related to the existing text-only warnings in the U.S. to provide a comparison for follow-up measures. Participants also provided an expired carbon monoxide (eCO) reading at baseline to confirm recent cigarette smoking. At the conclusion of the baseline session, the trial exposure was implemented by affixing pictorial warning labels covering 50% of the front and back of the cigarette pack(s) that participants brought to the baseline session and indicated they would use the following day.

On day two, participants responded to two mobile phone text messages assessing the number of cigarettes they smoked that day and motivation to quit [18]. For the purpose of the study, a third text message was administered asking participants to provide a picture of their cigarette pack with the pictorial warning label visible to confirm adherence to this aspect of the study protocol. On day three, participants completed a follow-up interview similar to baseline with questions about perceptions and behaviors in response to the study pictorial warnings [16,17]. Participants also completed a scale that was validated in a previous study to capture acceptability of the study protocol [19].

We conducted descriptive statistical analyses to assess the feasibility of the protocol, including retention, adherence, and responses to baseline, text messaging, and follow-up measures. We also examined changes in perceptions and behaviors reported at baseline in reference to the existing text-only warnings and at follow-up in reference to the study pictorial warning descriptively and using paired *t*-tests. The Georgetown University Institutional Review Board approved the study protocol, and all participants provided written informed consent to participate.

## Results

**Figure 1:**
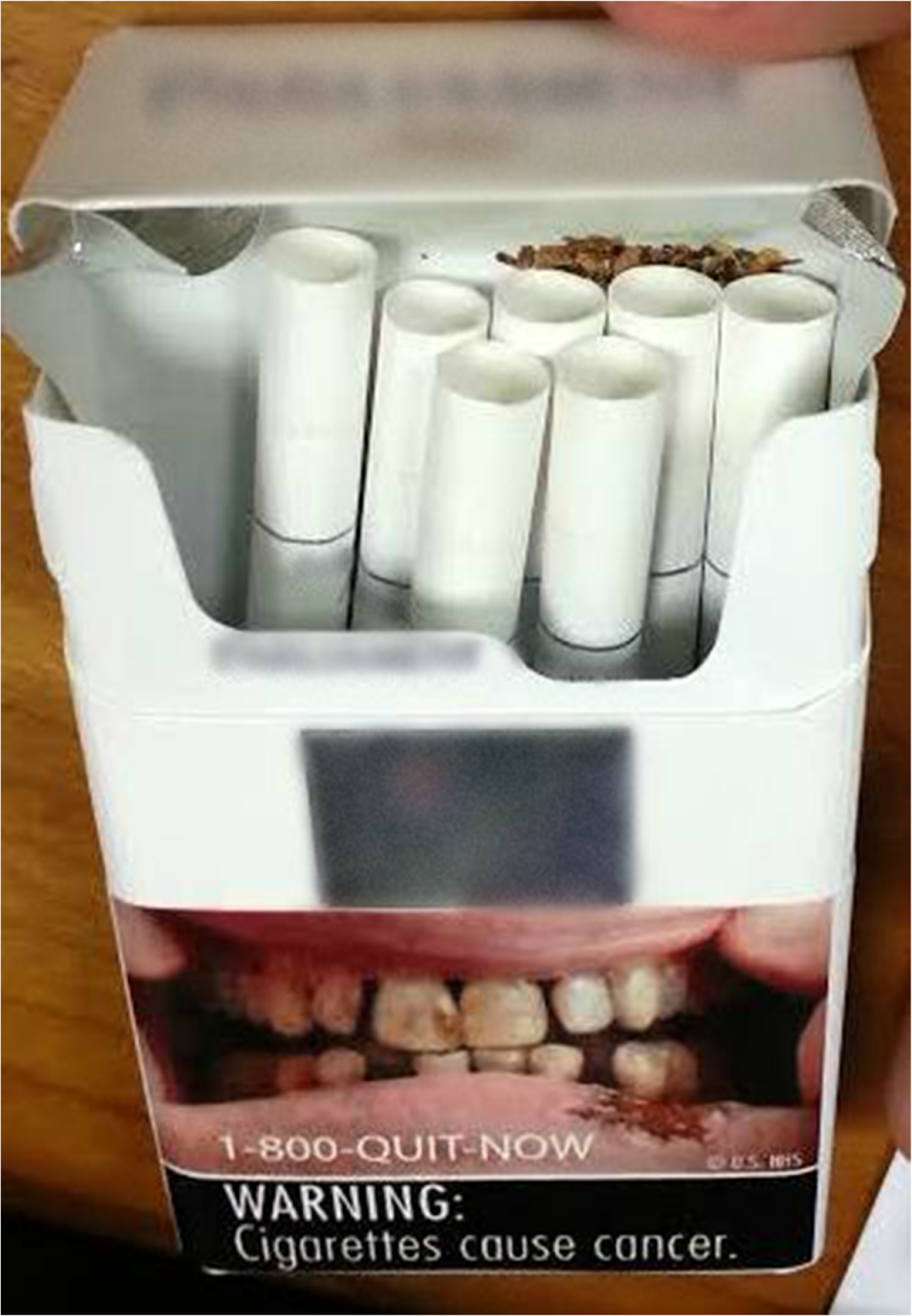
Sample participant cigarette pack photograph with study warning label.

Characteristics of the sample are shown in Table 1. At baseline, all participants provided an eCO reading verifying their smoking status (Table 1) and successfully completed a test of the text messaging procedure. On day two, we received responses to 100% of the text message assessments, including message replies with photographs of participants’ cigarette packs with study warning labels to verify adherence (Figure 1). On the scale designed to capture protocol acceptability administered at follow-up, participants indicated the protocol was acceptable (*M* 37.3, *SD* 3.4, range 8–40).

**Table 1:** Sample characteristics at baseline

	***n*** **= 10**
Gender	
Male	6
Female	4
Age [*M (SD)*]	20.6 (2.1)
Race	
White	6
Other minority	4
Education	
College degree or greater	1
High school/Some college	9
Employment	
Part time	9
Student	1
Household income	
> $US 50,000/year	6
≤ $US 50,000/year	4
Cigarettes smoked per day [*M (SD)*]	5.6 (3.5)
Daily smoker	
Smoke every day	8
Smoke some days	2
Expired carbon monoxide [*M (SD)*]	5.7 (3.4)
Motivation to quit [*M, SD,* range 1–4]	2.5 (0.5)
Preferred cigarette brand	
Marlboro or camel	6
Another brand	4

Comparisons of participants’ perceptions and behaviors related to warnings at baseline and follow-up are shown in Table 2. Compared with baseline assessments of the existing text-only warnings in the U.S., participants were more likely to report the study pictorial warnings were noticeable (*p* = .013) and conveyed the health risks of smoking (*p* = .006). Participants were also more likely to indicate they noticed the warnings and warnings stopped them from smoking (both *p’s* = .052).

**Table 2:** Comparison smoking behaviors and perceptions and behaviors related to cigarette warning labels

	**Baseline**	**Follow-up**	**Average change**	***P*-value**
Perceptions of Warnings (range 1–5)				
Warnings are Noticeable	2.7 (1.2)	4.1 (1.3)	1.40 (1.42)	.013
Warnings are Serious	3.8 (1.1)	3.9 (1.3)	0.0 (1.87)	1.00
Warnings are Believable	4.4 (0.7)	3.9 (0.9)	−0.50 (0.84)	.096
Warnings Convey Risks	2.2 (1.1)	3.5 (1.3)	1.13 (1.16)	.006
Warning Behaviors				
Notice Warnings (range 1–5)	3.4 (1.0)	3.9 (1.3)	0.50 (0.71)	.052
Read Warnings (range 1–5)	3.1 (1.0)	3.0 (1.3)	−0.10 (0.99)	.757
Warnings Stopped you From Smoking (range 1–4)	1.1 (0.3)	1.6 (0.8)	0.50 (0.71)	.052

Note: Data are displayed as mean (standard deviation). Questions at baseline assessed perceptions and behaviors in reference to existing text-only cigarette warnings in the U.S. Questions at follow-up assessed perceptions and behaviors in reference to the pictorial warnings participants used during the study.

## Discussion

This study used a behavioral epidemiology framework [8,9] to conduct a pilot trial of a method to determine the impact of pictorial warning labels among smokers. The study extends methods that have been used in prior research examining plain cigarette packaging [11-13] to investigate the impact of pictorial cigarette warning labels in a real-world manner. Consistent with these earlier studies, the findings indicate that a research protocol combining multi-modal behavioral assessments including self-report interviews, biochemical verification of cigarette smoking, and mobile phone text messaging is a feasible approach in this research context. In the future, research adopting trial-like methods such as those used in this study can aid in understanding the impact of pictorial warnings on smokers’ behaviors and provide evidence that can inform the implementation of pictorial warning labels in various settings. However, given the small sample size and pilot nature of this work, additional studies are needed to further validate the results.

Our results with respect to sample retention, protocol adherence, and participants’ favorable views towards acceptability of the study methods support the feasibility of the methods. The comparisons of participants’ perceptions and behaviors related to existing text-only warnings at baseline and the pictorial warnings used during the study at follow-up is also consistent with the body of evidence demonstrating that pictorial warnings are more likely to capture smokers’ attention, raise awareness of the health risks of smoking, and influence other smoking-related outcomes compared with text-only warnings [1,3]. Study participants perceived pictorial warnings to be more noticeable and to more effectively convey health risks of smoking, and reported that pictorial warnings were more likely to stop them from smoking compared with existing text-only warnings in the U.S. These findings lend further support to the feasibility of the approach for capturing relevant outcomes, however they should be considered in light of notable limitations to the study.

Given the pilot nature of this work and the small convenience sample comprised of intermittent smokers, the statistical results should be interpreted cautiously. This pilot study also was not designed to assess the impact of warnings on changes in smoking behaviors (e.g., cigarette smoking, quit attempts) with adequate power or sufficient duration of follow-up. We relied on self-report measures, which could be influenced by biases such as social desirability. Future research can build from this study in several important ways. The sample was comprised of young adults who smoked few cigarettes per day on average, and research to investigate the feasibility of using these methods among heavier smoking populations is warranted. Future studies can also extend the results of this work by using longer follow-up periods to assess warning label impact on smoking behaviors, as well as experimental research designs to examine manipulations to warning label design and other packaging regulations (e.g., plain cigarette packaging) to inform policy decisions.

## Abbreviations

FCTC: Framework convention on tobacco control; Tobacco Control Act: Family smoking prevention and tobacco control act; FDA: U.S. food and drug administration; eCO: Exhaled carbon monoxide.

## Competing interests

The authors declare that they have no competing interests.

## Authors’ contributions

DM, SEM, ACJ, and KPT made substantive contributions to the conception and design of the study; data acquisition, analysis, and interpretation; and preparation of the manuscript. JDK made substantive contributions to data interpretation and preparation of the manuscript. All authors read and approved the final manuscript.
